# Knowledge gaps in the assessment of antimicrobial resistance in surface waters

**DOI:** 10.1093/femsec/fiab140

**Published:** 2021-10-08

**Authors:** Magdalena Niegowska, Isabella Sanseverino, Anna Navarro, Teresa Lettieri

**Affiliations:** European Commission, Joint Research Centre (JRC), Via Enrico Fermi 2749, 21027 Ispra, Italy; European Commission, Joint Research Centre (JRC), Via Enrico Fermi 2749, 21027 Ispra, Italy; European Commission, Joint Research Centre (JRC), Via Enrico Fermi 2749, 21027 Ispra, Italy; European Commission, Joint Research Centre (JRC), Via Enrico Fermi 2749, 21027 Ispra, Italy

**Keywords:** antimicrobial resistance (AMR), antibiotic resistance genes (ARG), antibiotic resistant bacteria (ARB), co-resistance, water environment, metagenomics

## Abstract

The spread of antibiotic resistance in the water environment has been widely described. However, still many knowledge gaps exist regarding the selection pressure from antibiotics, heavy metals and other substances present in surface waters as a result of anthropogenic activities, as well as the extent and impact of this phenomenon on aquatic organisms and humans. In particular, the relationship between environmental concentrations of antibiotics and the acquisition of ARGs by antibiotic-sensitive bacteria as well as the impact of heavy metals and other selective agents on antimicrobial resistance (AMR) need to be defined. Currently, established safety values are based on the effects of antibiotic toxicity neglecting the question of AMR spread. In turn, risk assessment of antibiotics in waterbodies remains a complex question implicating multiple variables and unknowns reinforced by the lack of harmonized protocols and official guidelines. In the present review, we discussed current state-of-the-art and the knowledge gaps related to pressure exerted by antibiotics and heavy metals on aquatic environments and their relationship to the spread of AMR. Along with this latter, we reflected on (i) the risk assessment in surface waters, (ii) selective pressures contributing to its transfer and propagation and (iii) the advantages of metagenomics in investigating AMR. Furthermore, the role of microplastics in co-selection for metal and antibiotic resistance, together with the need for more studies in freshwater are highlighted.

## Teaser

The spread of antibiotic resistance in water environments is a complex issue affected by knowledge gaps relative to the (i) selection pressures, (ii) lack of harmonized assessment and (iii) methodological approaches, which require intense research efforts to estimate and limit the risk to human health.

## INTRODUCTION

The widespread use and misuse of antibiotics, especially through direct use in healthcare practice and in food animal production, along with the release of their residue concentrations from wastewaters to the environment represent major contributors to the acquisition and spread of antibiotic resistance (WHO 2014; O'Neill 2015; Kraemer, Ramachandran and Perron 2019; Polianciuc *et al*. 2020). With 700.000 deaths resulting from infections unresponsive to antibiotic therapies every year, the World Health Organization (WHO) included this phenomenon among the main threats to global health (WHO 2018a and 2020a) and the number is estimated to reach 10 million by 2050 if no action to combat antimicrobial resistance (AMR) is taken (UN 2019). AMR is defined as the ability of a microorganism to withstand the effects of antimicrobial agents, including antibiotics and other compounds, that were initially able to treat the infection caused by that microorganism (WHO 2020b). Here, the acronym AMR is used when referring to a general effect of antibiotics and substances discussed further, while ‘antibiotic resistance’ refers to studies investigating resistance to antibiotics only when such term was specifically used in the source publication.

Lately, AMR has been defined a ‘slow moving pandemic’ with enormous impact for the society, the consequences of which are nowadays underestimated due to long time necessary to reach the maximum extent (NPR 2020). Over recent years, excessive and improper use of antibiotics has been addressed by the European Commission first (EC COM(2011)748) and other international authorities (WHO 2014; O'Neill 2016) with emphasis on global surveillance of antimicrobial consumption in humans and animals. Further, One Health European strategies and joint recommendations of WHO, Food and Agriculture Organization (FAO) of the United Nations and World Organization for Animal Health (OIE) highlighted the environmental contribution to AMR, particularly the role of polluted waterbodies (EC COM (2017) 339; WHO, FAO and OIE 2020).

At European level, the amended Water Framework Directive (WFD) includes the Watch List (WL) program which aims to gather good data quality for substances contaminating waterbodies to determine the risk they may pose to the aquatic environments (EC 2018). Given the threat on public health posed by the spread of AMR through waterbodies, three antibiotics belonged to the macrolide class (azithromycin, clarithromycin, and erythromycin) were introduced in the first WL (Calvalho *et al*. 2015), followed by two additional antibiotics (amoxicillin, ciprofloxacin) in the revised WL (Loos *et al*. 2018) and more recently sulfamethoxazole, trimethoprim and other antimicrobials (azole antifungal agents) have been included in the 3^rd^ WL (Gomez Cortes *et al*. 2020; EC 2020a). The latter are substances either used as pharmaceuticals or pesticides which raised concern for their contribution to AMR spreading (Hendrickson *et al*. 2019). To minimize excessive and inappropriate use of antimicrobials in animal and human healthcare, a 50% reduction of their sales for livestock and aquaculture by 2030 is one of the EU objectives within Farm to Fork Strategy (EC 2020b), while development of alternatives or novel antimicrobials, review of legislation to restrict and optimize their use along with non-legislative measures against AMR are foreseen by the Pharmaceutical Strategy for Europe (EC 2020c). In addition, expanded environmental monitoring and improved environmental risk assessment of medicinal products, including antibiotics and ARG, are among ongoing actions under the EU Strategic Approach to Pharmaceuticals in the Environment (EC 2019a).

Antibiotics enter surface water compartments mainly with hospital and urban wastewaters, industrial wastewaters (in particular from pharmaceutical production facilities), leachate from waste disposal sites, agricultural runoff from fields fertilized with manure and aquaculture practice (Baquero, Martínez and Cantón 2008; Kraemer, Ramachandran and Perron 2019). Among natural, semi-synthetic and synthetic antibiotics, many are poorly biodegradable and contribute to the spread of AMR by persisting in the environment (Ingreslev and Halling-Sørensen 2000; Kümmerer 2009; Adamek, Baran and Sobczak 2016; Reis *et al*. 2019; Rodriguez-Mozaz *et al*. 2020). For example, erythromycin has been considered not readily biodegradable raising a very high level of concern reagrding persistance (UBA 2014). Amoxicillin, which is the most widely used semi-synthetic β-lactam antibiotic recommended as first or second line therapy for common infection diseases, has a half-life in water of ∼20 days at pH 7 (Loos *et al*. 2018; WHO 2018b). However, a high consumption of more degradable antibiotics (e.g. β-lactams which are highly unstable in the environment) determines constant emissions of antibiotic residues and their metabolites by wastewater treatment plants (WWTP) at faster rates than their environmental removal. This results in the continuous presence of antibiotics at low concentrations in water environments where they can be degraded to metabolites that retain antimicrobial activity. Moreover, it has been shown that conventional wastewater treatment processes remove antibiotic resistant bacteria (ARB) and antibiotic resistance genes (ARGs) at limited efficiencies, leading in certain cases to their increase in final effluents (Ferreira *et al*. 2006 and 2007; Zhang, Zhang and Fang 2009; Łuczkiewicz *et al*. 2010; Novo *et al*. 2013; Rodríguez-Chueca *et al*. 2019). This issue has been raised in the evaluation of the Urban Waste Water Treatment Directive (UWWTD) and follow up actions including possible upgrading of WWTP to more advanced technologies as well as revision of the UWWTD are expected (EC 2019b).

ARB constitute a potentially unlimited source of ARGs which can be replicated and disseminated among connected aquatic systems and organisms worldwide even when the selective pressure from environmental antibiotic residues disappears (Salyers and Amábile-Cuevas 1997; Pruden, Arabi and Storteboom 2012). The risk of clinically relevant ARG transfer from water environments to animal or human pathogens and *vice versa* has been widely hypothesized, especially in relation to impacted settings and resistance to last resort antibiotics (Cabello *et al*. 2016; Jiang *et al*. 2017; Marathe *et al*. 2017; Santos and Ramos 2018; Ben *et al*. 2019). However, it is uncertain to which extent this risk is due to various environmental pathways of discharged antibiotics (Bengtsson-Palme, Kristiansson and Larsson 2017).

Despite limited studies estimating daily intake of antibiotics through food consumption (Ben *et al*. 2019) and the documented presence of antibiotics, ARB and ARGs in the human gut (Casals-Pascual, Vergara and Vila 2018), the question of ARG spread and its impact on human gut microbiome has not been sufficiently addressed. Also, the spread of ARGs through the application of manure as soil fertilizers and their further transfer to surface waters is complex and needs more accurate estimates (Heuer, Schmitt and Smalla 2011; Udikovic-Kolic *et al*. 2014; Topp *et al*. 2018).

Although a large body of scientific literature reports the presence of antibiotics in surface waters and studies focused on genes involved in AMR, mechanisms leading to the selection and acquisition of resistance determinants by bacterial strains as well as ARG maintenance in aquatic environments remain elusive. At policy level, no safety threshold values or standardized guidelines on the environmental risk assessment of antibiotics currently exist, which is largely due to insufficient data on environmentally relevant concentrations (Schmitt, ter Laak and Duis 2017). Here, we briefly discuss the risk assessment of AMR in aquatic environments as a link between the acquisition of resistance by sensitive bacteria and risk to human health. In particular, we focus on areas characterized by low anthropogenic impact and on the application of metagenomics for environmental AMR detection. We also review the current state-of-the-art and knowledge gaps related to the relationship between the concentrations of antibiotics and the acquisition of ARGs by antibiotic-sensitive bacteria, paying attention to the unknowns involving the impact of heavy metals and other indirect selective agents on AMR.

## RISK ASSESSMENT AND BIOAVAILABILITY OF ANTIBIOTICS IN AQUATIC ENVIRONMENTS

In the WFD, the assessment of priority substances and other substances of concern is based on environmental quality standards (EQS) referring to concentrations established for single chemicals at which adverse effects in humans and aquatic wildlife may be elicited (EC 2000). This parameter, however, translates only the toxicological potential of a compound without considering the spread of ARGs among microorganisms and underestimating the threat of resistant infections. The need to include AMR in the risk assessment was envisaged in the 3^rd^ WL program, as for the sulfamethoxazole and trimethoprim, the predicted no-effect concentrations (PNEC) based on the minimum inhibitory concentrations (MIC), were additionally evaluated to be proposed as safety thresholds (Bengtsson-Palme and Larsson 2016). However, these values were only selected if lower than the PNEC value, based on ecotoxicological data, to be more protective towards the aquatic ecosystem (Gomez Cortes *et al*. 2020).

According to antibiotic concentrations reported in Europe, mean values in surface water are equal or close to 0.01 µg L^–1^ for most antibiotics, however levels from less than 0.001 µg L^–1^ to over 1 µg L^–1^ were also observed (Sanseverino *et al*. 2019). At the same time, antibiotic concentrations in WWTP effluents worldwide ranged from 0.002 to 1.4 µg L^–1^ (Sanseverino *et al*. 2019). The European Medicines Agency (EMA) recommends a tiered approach to assess ecotoxicological risk from pharmaceuticals in surface waters with concentration threshold of 10 ng L^–1^ derived from predicted environmental concentration (PEC) proposed as safety limit (except for substances such as endocrine disruptors or antiparasitics eliciting effects in biota at lower concentrations) (EMA 2018). Two-phase toxicity testing procedure, including Organization for Economic Cooperation and Development (OECD) growth rate bioassays in cyanobacteria suggested for antibiotics, should be performed entirely to identify medicinal products which may affect organisms. Similar to EQS, these guidelines do not refer to the limitation of AMR which can occur in the presence of even lower antibiotic concentrations in water environments (Chen *et al*. 2017; Oberlé *et al*. 2012).

In several recent studies, the potential risk of antibiotics on aquatic organisms and AMR selection has been evaluated using risk quotient (RQ) method according to the EMA guideline, based on the ratio between PEC or measured effect concentration (MEC) and predicted no effect concentration (PNEC), the latter only derived from ecotoxicological data (EMA 2018). Despite low RQ in main Chinese rivers and coastal areas, the risk from some antibiotics was estimated to be significant due to their persistence resulting from high usage and inefficient removal, with algae particularly affected by sulfadiazine (Chen *et al*. 2017; Qiu *et al*. 2019; Zhang *et al*. 2012). This approach was adopted to evaluate risk at three trophic levels including algae, invertebrates, and fish in Hong Kong rivers, where antibiotics exerted major impact on algal species (Deng, Li and Ying 2018).

To consider effects from antibiotic mixtures, Chen *et al*. (2018) estimated the toxicity to cyanobacteria and microalgae that may arise from synergistic action of four antibiotics based on concentrations detected in environmental samples, highlighting the increased risk compared to substances assessed individually (Hossain *et al*. 2017). In few studies, ecological risk from selected antibiotics has been reported for primary producers in aquaculture environments based on short-term exposure data (Bengtsson-Palme and Larsson 2016; Quinlan *et al*. 2011; Sun *et al*. 2016).

As PNEC values inform about the adverse effects, such as growth inhibition, elicited in organisms by environmental pressure, RQ has been used by some authors to evaluate fractions of bacteria with acquired resistance. Enrofloxacin, ampicillin and levofloxacin showed to pose a high risk of AMR by microbial communities in an experimental aquaculture pond including target and non-target species (Hossain *et al*. 2017). A dose-series exposure of freshwater periphyton to tetracycline in an experimental mesocosm mimicking natural stream conditions proved that antibiotic resistance and significant changes in the biotic community are induced within few days by concentrations of the antibiotic currently observed in aquatic environments (0.5 μg L^–1^) (Quinlan *et al*. 2011). In a literature-based prediction comprising data on MIC, PNEC values were estimated for a wide range of antibiotics at levels from 8 ng L^–1^ to 64 μg L^–1^ (Bengtsson-Palme and Larsson 2016).

The association between levels of resistance detected in water and environmental occurrence of antibiotics may be affected by site-specific factors and by intrinsic characteristics of the compound, therefore RQ may not be true for the same antibiotic in different scenarios. Variation of environmental conditions, such as salinity and pH, and physicochemical properties of antimicrobial molecules may influence the bioavailability of antibiotics, e.g. by determining changes in sorption capacity to decomposing organic matter (Xu and Li 2010; Zhang *et al*. 2018). A large body of evidence indicates that the co-occurrence of multiple chemicals, such as antibiotics and metals, in waterbodies and the presence of mobilizable ARGs (e.g. in plasmids or transducing phage particles) promote the AMR development (Lood, Ertürk and Mattiasson 2017; Schmitt, ter Laak and Duis 2017). Finally, biological features typical to each bacterial species differentially regulate their exposure and susceptibility to bioactive compounds through biofilm formation or complex cell-to-cell communication networks (Schroeder, Brooks and Brooks 2017).

Although no indications for ARG monitoring in environmental waters are currently in place (FAO 2019), discussions of the Working Group Chemicals under the WFD are at good progress. Inclusion of antibiotics and ARGs for monitoring in soil has been achieved thus far within the Land Use and Coverage Area frame Survey (LUCAS) under the EU Strategic Approach to Pharmaceuticals in the Environment (EC 2020c).

Main knowledge gaps underlying AMR spread not only refer to the relationship between antibiotics and ARGs as self-replicating elements but also to the abundance of resistant pathogenic bacterial species, their sources and behavior in the environment (FAO 2019). Risk assessment methods must therefore specifically consider a type of microbial community present in a waterbody and ARGs showing different rate of transmission that correlates with co-occurrence of pollutants or their transformation products. In this context, AMR surveillance in the water environment should be strictly linked to chemical monitoring which requires development of standardized protocols allowing interlaboratory comparison of results and reduction of possible technical errors.

## CORRELATION BETWEEN ANTIBIOTIC CONCENTRATIONS AND ARG

Despite evidence linking the transfer of ARGs between clinical settings and environmental compartments subjected to anthropogenic pressure, a real correlation with antibiotic concentrations is missing (Ben *et al*. 2019). Numerous studies reporting the presence of antibiotics and ARGs in environmental samples do not describe actual resistance of bacteria verified by experimental exposure. On the other hand, bacteria able to grow on culture media supplemented with antibiotics are not always screened for the acquired ARGs using culture-independent methods as a confirmatory step of resistance observed *in vitro*.

Culture-independent methods, mainly PCR-based and high throughput sequencing, provide means to assess genetic material by detection and/or quantification of ARGs from entire microbial pool including unculturable microorganisms but do not consider their functional capability (Luby *et al*. 2016; Vaz-Moreira, Nunes and Manaia 2014). *In vitro* testing can overcome this gap only in a remarkably small fraction of microbial species capable of growing under laboratory conditions which does not necessarily reflect the real scenario (Allen *et al*. 2010). Indeed, numerous species and genotypes present in complex natural communities may be markedly sensitive to antibiotics with respect to pure strains used in laboratory experiments and therefore taken over by more tolerant bacteria (Zhang *et al*. 2011). In this context, antibiotic concentrations able to promote AMR under laboratory conditions, which are lower than those inhibiting bacterial growth (Andersson and Hughes 2012), may not correspond to levels eliciting the same effect in the environment.

In a study combining culture-dependent and culture-independent approaches, culturable bacteria bearing plasmid-mediated resistance genes to antibiotics frequently used in marine aquaculture were isolated (Buschmann *et al*. 2012). Nonetheless, the examined antibiotics could not be detected in the counterpart sediment samples, probably due to biodegradation or water currents causing a high dispersal of materials (Buschmann *et al*. 2012). In water samples from impacted sites, a significant correlation between antibiotic concentrations, resistant bacteria and sulfonamide resistant dihydropteroate synthase (*sul1*) gene variant conferring resistance to sulfonamide antibiotics was found when comparing raw wastewater influent and a final effluent (Gao, Munir and Xagoraraki 2012). The drop of sulfonamides concentrations throughout the WWTP from initial 1535.9 ng L^–1^ to 261.1 ng L^–1^ corresponded to almost 3 log reduction of colony-forming units (CFU) of bacteria screened on antibiotic-selective culture media (3.09×10^3^ CFU/mL in final effluent) and to 2 log decrease in *sul1* gene abundance quantified by real-time PCR. This example underlines the potential of ARGs and bacteria carrying these resistance genes to persist despite adverse conditions such as wastewater treatment processes and to be dislocated by natural forces like water currents.

Thus far, combined analysis describing a correlation between the concentrations of antibiotics and resistance genes has been performed in few studies (Yang *et al*. 2018). Inability to find significant correlations may be due to several factors. It has been partially explained by changes in the prevalence of resistance which is developed at generally very slow rates compared to the use of antibiotics which can follow rapid variations.

In aquatic ecosystems, antibiotics and ARG are subject to differential environmental fate. While events such as degradation through hydrolysis and photolysis, sorption to sediments and organic substances or dispersion through transport may affect the limit of detection of antibiotics, the rate of ARG decay following a similar fate may differ (Nnadozie and Odume 2019). Diverse environmental matrices (i.e. surface water, sediments, soil from a drawdown area) are also determinant in shaping ARG dynamics either for genes common to all or for those specifically found in a given habitat (Chen *et al*. 2019). Moreover, the selection for ARGs can be influenced by chemical pollutants, in particular heavy metals and polycyclic aromatic hydrocarbons (PAH), which exert a selective pressure for the occurrence of ARGs in the environment (Nguyen *et al*. 2019; Wang *et al*. 2017a). Finally, methods used for the detection of ARGs are rarely uniform in published literature and hardly focus on all available gene variants conferring resistance to a single antibiotic. Instead, testing methods usually include only selected ARGs that may not be representative of all gene variants within the population. Establishing such relationships needs therefore a holistic assessment to identify possible paths that ARGs follow starting from the source of antibiotics’ release to the environment which is the main determinant of downstream genetic AMR pool.

## ENVIRONMENTAL CONCENTRATIONS OF ANTIBIOTICS AND STUDIES ON LOW-IMPACT AREAS

**Figure 1.**
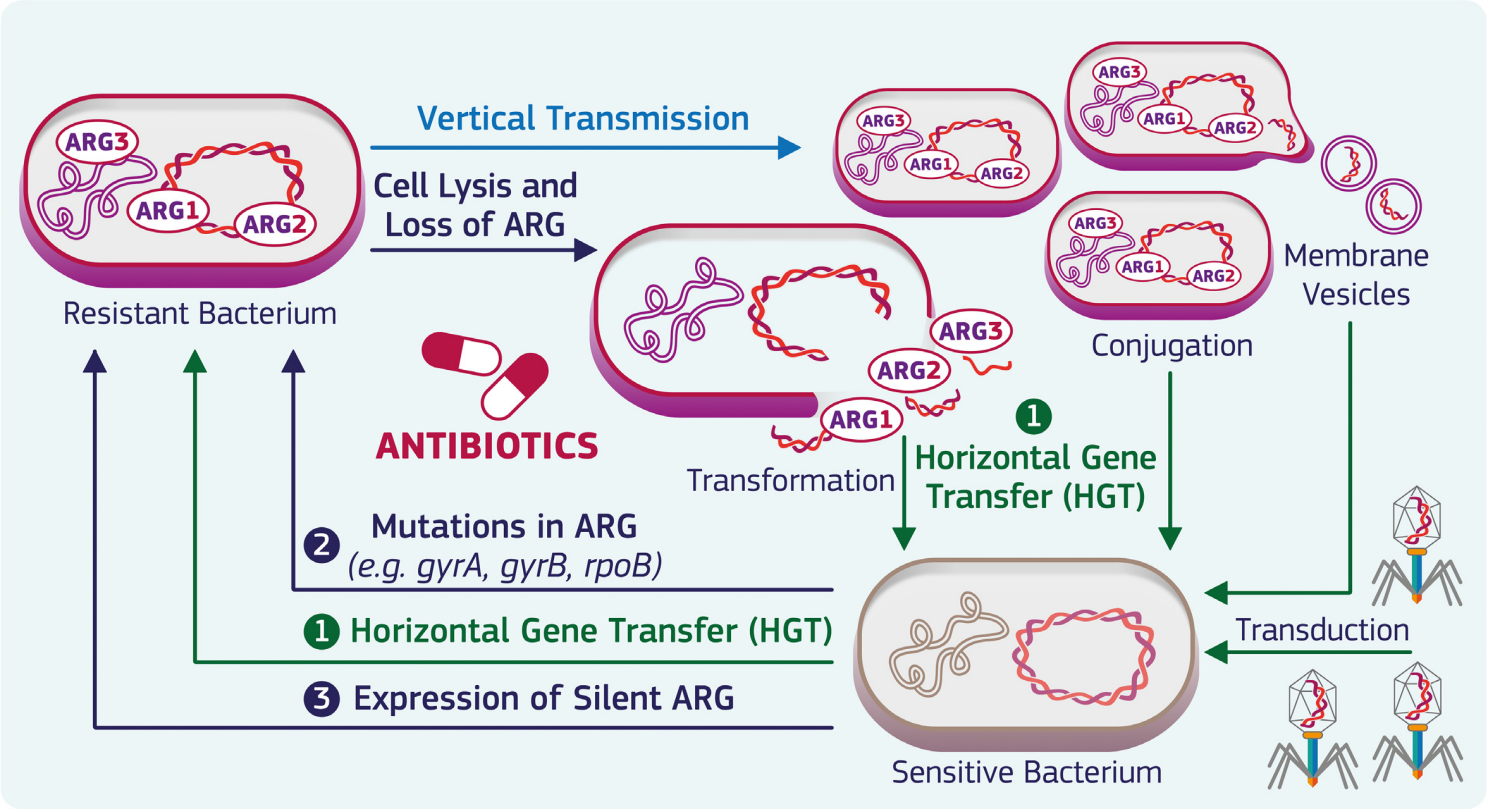
Synergistic effects of antibiotics and ARGs on the spread of AMR. ARGs can be acquired by sensitive bacteria following exposure to antibiotics present in the environment. This may occur through several pathways such as 1. HGT through conjugation with already resistant bacteria, transformation with plasmids or membrane vesicles carrying ARGs, transduction by phages and/or uptake of free-floating extracellular DNA; 2. Mutations selecting for resistance in genes encoding key bacterial proteins which are antibiotic targets (e.g. mutations in *gyrA* and *gyrB* which are responsible for resistance to fluoroquinolones, and mutations in *rpoB* which confer resistance to rifampicin) while other genes may encode enzymes able to actively degrade antibiotics (e.g. β-lactamases); and 3. Expression of silent resistance genes. Much more severe pressure due to antibiotic use in human and veterinary medicine also results in the expression of resistance in exposed microorganisms. ARGs released from lysed cells may be uptaken from waterbodies by sensitive microorganisms or those already bearing other resistance genes, in the latter case leading to multidrug resistance. Vertical gene transfer due to the proliferation of resistant bacteria further contributes to the spread of AMR.

The investigation of natural resistance is crucial to establish the abundance of ARB and ARGs in the environmental background as a reference point to understand the extent to which anthropogenic impact contributes to the ARG spread. Even at low concentrations, antibiotic pressure can lead to the acquisition and expression of ARGs (Murray *et al*. 2021) which increase in abundance along with the proliferation of resistant bacteria and can be exponentially propagated in the environment as resistance to single or multiple antibiotics by numerous microbial species (Fig. 1). At the same time, ARGs can be released from bacteria due to the lysis of dead cells and secretion from live cells in membrane vesicles or packed within phages constituting the extracellular ARG pool (Dong *et al*. 2019; Woegerbauer, Bellanger and Merlin 2020; Zhang *et al*. 2018a). In turn, some of these ARGs can be uptaken by competent bacteria through natural transformation or phage-assisted DNA transfer leading to AMR (Woegerbauer, Bellanger and Merlin 2020).

Antibiotic resistance can also be induced by mutations selecting for resistance and expression of silent resistance genes (Enne *et al*. 2006; Lupo, Coyne and Berendonk 2012; Woodford and Ellington 2007). These mechanisms have recently been discussed in a European technical report on the contribution of water to AMR, also providing relative concentrations of antibiotics detected in samples from surface waters worldwide (Sanseverino *et al*. 2019). Similarly, lists of ARGs identified in diverse types of environmental matrices are available (Zhang *et al*.^2009^; Zhuang *et al*. 2021).

Even though the presence of antibiotics and ARGs in anthropogenically impacted environments is already widely discussed, the phenomenon of AMR spread to non-impacted areas carries many unknowns and is poorly described (Hooban *et al*. 2020; Smalla *et al*. 2018). Studies on these areas are challenging due to the background resistance which microorganisms develop upon exposure to various stimuli (e.g. nutrient status, hypoxia) and to the naturally-occurring resistance such as *i)* intrinsic resistance independent of any selective pressure and not acquired by horizontal gene transfer (HGT) (Cox and Wright 2013), or *ii)* silent resistance genes able to confer AMR only when expressed in other than their native hosts (Dantas and Sommer 2012). Further, antibiotic-producing bacteria contribute to the natural level of antimicrobial metabolites (e.g. streptomycin, aminoglycosides, β-lactams) in the environment, bearing in turn specific ARGs as self-defense mechanisms (Al-Amoudi *et al*. 2016; D'Costa *et al*. 2011; van der Meij *et al*. 2017). For example, nitric oxide can induce resistance to aminoglycosides by blocking aerobic respiration and energy-dependent phases of antibiotic uptake (McCollister *et al*. 2011). Consequently, pollution by ARB can be considered when increased fraction of resistant bacteria is observed with respect to normal levels of natural resistance.

It is recognized that remote regions may bear evidence of AMR derived from human activities due to transport of ARB and ARGs in the environment contributing to the conversion of pristine habitats into low-impact areas. Such trends have been demonstrated by detection of human-specific mitochondrial gene target in Arctic marine sediments (Tan *et al*. 2018) and poor correlation of ARGs and class 1 integrases (*intI1)* genes with the degree of anthropogenic impact (Zhang *et al*. 2020). When evaluating the association between antibiotic concentrations and ARGs in sediments from sites along Poudre River (United States of America) subjected to different anthropogenic pressures, bacteria resistant to tetracyclines and sulfonamides were found in the low-impact area at the river source devoid of antibiotics (Pei *et al*. 2006). The numbers of ARB increased downstream proportionally to the impact of urban and agricultural activities. Similar observations were made based on metagenomics profiles from samples along the Yarlung Tsangpo River (Tibetan Plateau), where ARGs present already at the source, mainly *bacA* conferring resistance to bacitracin, increased with the human activities (Liu *et al*. 2021a). Additionally, abundant ARGs showed lower diversity, were less prone to dispersal compared to rare gene targets and differently impacted by the land use, urbanization and dam construction (Liu *et al*. 2021b). The two last activities increased the presence of genes conferring resistance to sulfonamide/tetracycline and chloramphenicol/aminoglycoside, respectively. In another study, ARGs were investigated among bacterial communities in freshwater sediment from the Patagonian island Tierra del Fuego characterized by extremely low degree of urbanization (Nardelli *et al*. 2012). The outcomes revealed the presence of class 1 integrons grouped within the most successful elements involved in acquisition, maintenance and spread of AMR in clinical settings (Fluit and Schmitz 2004; Hall and Collis 1998; Mazel 2006). Similar results were obtained from environments hardly affected by any kind of human activity such as deep terrestrial subsurface where 90% of isolates carried ARGs to at least one antibiotic (Brown and Balkwill 2008). Other remote areas including Antarctic soils, glacier lakes and deep ocean sediments showed the presence of ARG, although different from genes conferring resistance in urban or agricultural areas, suggesting that a historical ARG reservoir evolved as a mechanism of microbial adaptation to face the selective pressures of the environment (Chen *et al*. 2013 and 2016; Patel and Rosenthal 2007; Van Goethem *et al*. 2018; Wang and Sun 2017). In line with these findings, antibiotic resistance has been detected in commensal bacteria of humans and animals inhabiting regions not subjected to antimicrobial use (Pallecchi *et al*. 2008).

A thorough investigation is needed to understand whether AMR detected in low-impact areas has natural background or if it results from the spread of ARG and resistant bacteria originating from areas impacted by human activities. Another challenge would be to estimate the impact of natural resistance on AMR propagation upon exposure to discharged antimicrobial compounds. Such assessment might be highly complicated by river flows and marine currents which contribute to dispersing introduced antibiotics, thereby lowering their levels to environmental concentrations. At the same time, more efforts should be dedicated to analyze the effects from environmentally realistic concentrations of antibiotics outside areas such as WWTPs which often reflect worst-case scenarios.

## AMR AND PRESSURE FROM ANTIBIOTIC METABOLITES AND HEAVY METALS

The distribution of antibiotics in surface water may not be representative of compartments where utilization and sorption of antibiotic residues is more intense due to different rates of degradation in the environment and excretion from exposed organisms. As demonstrated by numerous studies, partitioning properties determine uneven distribution between water and sediments (Chen and Zhou 2014; Lei, Lu and Liu 2015; Li *et al*. 2018) while different sorption strengths to organic or inorganic phase may hamper the extraction of total antibiotic amounts for analytical purposes (Schmitt, ter Laak and Duis 2017). For this reason, particular attention should be paid when selecting the type of environmental matrix for risk assessment.

Certain metabolites have as well been shown to influence AMR. The excretion rate of antibiotics in an unchanged form may reach 90% in human and animal urine (Schmitt, ter Laak and Duis 2017; Sarmah, Meyer and Boxall 2006) with the remaining fraction of metabolites that may contain bioactive moieties. The degradation processes of antibiotics in aquatic environments may result in the release of metabolites which retain antimicrobial activity at the same or even increased level compared to their parent compounds (Berkner, Konradi and Schönfeld 2014; Jaén-Gil *et al*. 2020; Majewsky, Glauner and Horn 2015). This is the case for transformation products of sulfamethoxazole (García-Galán, Silvia Díaz-Cruz and Barceló 2008), one of the most important antibiotics monitored in European surface waters (Sanseverino *et al*. 2019). Similar to antibiotics, a complete elimination of their metabolites through water treatment processes is not attained (Majewsky *et al*. 2014), which implies the need of monitoring the concentrations of bioactive substances that may be generated during physiological metabolization and natural degradation of antibiotics. However, such solutions are hardly achievable in real scenarios, reason for which monitoring of the most relevant degradation products may be the first step in this approach.

Alongside antibiotics, heavy metals from natural and anthropogenic sources are widely distributed in water environments. Elevated environmental exposure to metals has raised an ecological and public health concern linked to the effects of metal toxicity (Bradl 2005; EEA 2018). An increasing scientific evidence demonstrates the relationship between molecular strategies developed by microbial species to resist pressure from a range of metals, antibiotics and the spread of AMR (Imran, Das and Naik 2019; Komijani *et al*. 2021; Pal *et al*. 2017; Poole 2017). It has been shown that metal resistance and antibiotic resistance in microorganisms are governed by shared genetic mechanisms and can be encoded by the same genetic elements (Randall *et al*. 2015). Similar findings have been described for cross-resistance to antibiotics and biocides (e.g. triclosan) (Carey and McNamara 2015; Gilbert and McBain 2003; SCENIR 2009; Webber *et al*. 2017). A study assessing the link between antibiotic and metal resistance in marine bacterial isolates found that most resistance genes were chromosomally located and identified homologs of efflux pump genes which are commonly expressed to overcome pressure from heavy metals and may further contribute to resistant phenotypes (Lloyd, Nazaret and Barkay 2018). Similar results were obtained among bacterial communities sampled from hot springs (Jardine, Mavumengwana and Ubomba-Jaswa 2019). In these low-impact environments an intrinsic or plasmid-related resistance has been suggested to drive antibiotic resistance and heavy metal tolerance, failing however to find a relative association (Jardine, Mavumengwana and Ubomba-Jaswa 2019).

Microplastics have recently been described as drivers of co-selection for resistance to heavy metals and antibiotics among bacteria associated with plastic particles (Imran, Das and Naik 2019). This results *inter alia* from major exposure due to the adsorption of heavy metals and antibiotics on the microplastic surface, which can be further increased by biofilm (Brennecke *et al*. 2016; Liu *et al*. 2021; Wang *et al*. 2017b). A study investigating the adsorption of five antibiotics (sulfadiazine, amoxicillin, tetracycline, ciprofloxacin and trimethoprim) on various types of microplastics concluded that ciprofloxacin was most easily adsorbed to the assessed plastic materials with freshwater environment significantly increasing the adsorption capacity (Li *et al*. 2018). It has also been observed that the presence of metal ions such as Cu^2+^, Zn^2+^ and Cr^3+^ in aqueous solution enhances the adsorption of levofloxacin on polyvinyl microplastics (Yu *et al*. 2020). Moreover, microplastic-associated bacteria display a higher frequency of plasmid transfer compared to free-living bacteria or those in natural aggregates (Arias-Andres *et al*. 2018). In line with this finding, the abundance of *int1* gene indicative of mobile genetic elements (MGEs) in the microplastic-associated biofilm impacted by treated wastewaters increased proportionally to particle concentration, but not in the water surrounding the microplastic particles (Eckert *et al*. 2018). Likewise, microbial communities from marine microplastics displayed an average relative abundance of ARGs 5.69 times higher compared to seawater samples (Yang *et al*. 2019). Although microplastics provide surface allowing persistent colonization by environmental microorganisms and transport of potentially pathogenic ARB to new ecosystems (Shen *et al*. 2019), still a limited number of studies exist on their contribution to co-selection for resistance to heavy metals and antibiotics in the water environment. A major stability and persistence of heavy metals relative to antibiotics, which are more rapidly degraded in the environment, may constitute a long-term selective pressure enhancing AMR. In riverine microcosms, exposure to 50 and 100 μg L^–1^ of zinc or copper correlated with increased prevalence of bacteria intrinsically resistant to cefotaxime and kanamycin (Silva, Tacão and Henriques 2021). A paleontology study of pond sediments found that Zn concentrations and bioavailability correlated with the level of Zn tolerance, resistance to clinically relevant antibiotics and composition of culturable microbial isolates (Dickinson *et al*. 2019).

Considering the interconnection between confining soil and water environments, differentially impacted soils along the Savannah River (USA) were assessed for co-resistance in bacterial taxa (Thomas *et al*. 2020). Although ARG conferring vancomycin and multidrug resistance as well as metal resistance genes (MRG) were common to all sampling sites, their relative abundance was increased in areas impacted by metals and radionuclides compared to the reference low-impact site (Thomas *et al*. 2020). Yet, metal-rich matrices with co-occurring antibiotic residues, such as livestock manure and municipal WWTP, have been suggested to promote ARG transfer associated with higher ARG levels in comparison to clinical settings (Yuan *et al*. 2020).

Although intrinsic antimicrobial effects of some metals, in particular silver and mercury, may be elicited even at very low concentrations (Lemiere *et al*. 2013; Silver 2003), tolerance to exceptionally high concentration ranges was detected in non-urbanized areas, such as Antarctic marine waters and shallow sediments (10 mg L^–1^ for mercury, and varying for cadmium, copper, chromium and zinc depending on bacterial genus), and correlated with a double metal-antibiotic resistance in up to a 100% of samples (De Souza *et al*. 2006; Lo Giudice *et al*. 2013). Co-resistance driven by silver recently observed in hospital settings (Andrade *et al*. 2018) points at the risk of enhancing the phenomenon in aquatic environments through the dispersal of this metal into waterbodies that results from its broad use as a disinfecting agent.

Silver nanoparticles (Ag-NPs), employed in a wide range of biomedical and consumer products such as sunscreens, have been reported to induce AMR in some bacterial strains after repeated exposure (Kaweeteerawat *et al*. 2017) and expression of efflux pump genes associated with resistance against heavy metals in bacteria from environmental samples (Chen *et al*. 2019). Likewise, dosing with Ag-NPs correlated with increased abundance of integrons in experimental wastewater reactors (Ma *et al*. 2016) and was associated with HGT (Lu *et al*. 2020). Although results on Ag-NP-mediated co-resistance provided by most authors are contradictory, the Scientific Committee on Emerging and Newly Identified Health Risks (SCENIHR) highlighted that unrestricted use of Ag-NPs may pose environmental pressure resulting in future generation and spread of AMR (SCENIR 2014). An additional question to be explored regards the effects of Ag-NP colloidal solutions formed upon contact with other chemical compounds on AMR development (Bundschuh *et al*. 2018).

Even though the extent to which metals and related molecular mechanisms promote co-selection of antibiotic resistance is still unclear, it has been recently demonstrated that sub-inhibitory concentrations of heavy metals present in water environments facilitate mutations or horizontal transfer of ARG and are able to induce resistance to antibiotics at environmentally relevant levels (Andersson and Hughes 2012; Xu *et al*. 2017; Zhang *et al*. 2018b). In particular, exposure to 0.01 mg L^–1^ Cu(II), 0.01 mg L^–1^ Ag(I), 0.1 mg L^–1^ Cr(VI), and 0.05 mg L^–1^ Zn(II) was followed by downregulation of global regulatory genes (*korA*, *korB* and *trbA*) which resulted in increased expression of *trbB* and *traF* promoters by up to 5.1 folds (Zhang *et al*. 2018b). Proteins encoded by these two genes belong to the mating pair formation system involved in the establishment of conjugative bridges between cell surface proteins (Zatyka and Thomas 1998).

Such correlations appear stronger at short spatial scales and are lost with the distance from contamination source due to differences in fate of metals and antibiotics during transport (Kerrigan *et al*. 2018; Stepanauskas *et al*. 2006). Other studies reported antibiotic resistance to raise with decreasing concentrations of some heavy metals (lead, chromium, copper, zinc) present in the surrounding environment, although contrasting results have been obtained by different research groups (Seiler and Berendonk 2012; Zhou *et al*. 2015). In freshwater microcosms, 5 µg L^–1^ Cu concentrations increased the conjugative transfer 16 folds compared to control conditions (Wang *et al*. 2020).

Importantly, samples must be carefully selected for the assessment of metal-antibiotic synergistic actions. Sediments and microhabitats rich in organic matter (e.g. biofilm) attract metal deposition (Wright *et al*. 2006) meaning that concentrations measured in different matrices from the same site may show high variability.

## METAGENOMICS TO ADDRESS ENVIRONMENTAL AMR DETECTION

Metagenomics exploiting next generation technologies is a sequencing technique applied to analyze the entire genomic material from microbial communities with improved accuracy and deeper knowledge due to available databases. In environmental samples, this approach is useful in identifying and estimating relative gene abundance as a snapshot of microbial and genetic diversity including data relative to unculturable bacteria.

In recent years, metagenomics has been more frequently employed to investigate AMR in water environments giving a possibility to assess a large number of ARGs in one single experiment instead of performing multiple PCR/qPCR for each ARG one by one. Additional information can be obtained regarding the association with metal ion resistance (Reddy and Dubey 2019) and ways of ARG transmission by identifying key elements involved in gene mobilization in the entire bacterial community or in specific sample fractions, such as (MGEs) or phage genetic material (Chopyk *et al*. 2020; Moon *et al*. 2020; Pan *et al*. 2020; Raza *et al*. 2021). However, it is currently troublesome to compare the outcomes of various metagenomics studies due to complex data analysis and to the lack of standardized methods and protocols between different platforms.

Metagenomic techniques acquire more importance in ecosystems such as aquaculture sites and low-impact surface waters where effects of antibiotics on the prevalence and spread of ARG are less explored. When applied to aquaculture, metagenomics is becoming essential in evaluating the impact of current fish farming solutions on AMR in water ecosystems. The release of antibiotics in open aquaculture is of particular concern as they can be spread in the surrounding environment and persist over long periods in sediments (Amos *et al*. 2015; Rigos *et al*. 2004). It has been estimated that antibiotics administered to fish via supplemented feed are only partially absorbed (Cabello 2006) with largely unknown impact of released antimicrobials on non-target aquatic organisms and on the spread of resistance. Multi-trophic cultures of species representing different levels of the food chain (shrimp, selected fish species and/or duck) were found to increase the abundance of ARGs in water and sediments respect to fish monocultures (Xu *et al*. 2020; Yuan *et al*. 2019). Still, in sediments of a bullfrog farm, metagenomics analysis highlighted the association between plasmids carrying ARGs and resistance conferred to bacterial hosts against multiple antibiotics including classes not used in aquaculture (Chen *et al*. 2018). This approach permitted to associate the most dominant ARGs with bacterial genera as resistance carriers in mariculture (Jang *et al*. 2018) and to characterize the change of microbial community at the merge point of a river delta with backyard-based aquaculture effluents and at water/sediment interface in freshwater fish ponds (Nakayama *et al*. 2017; Xiong *et al*. 2015). In a recent study evaluating AMR in the crab aquaculture by metagenomics, distinct ARG distribution has been detected between water, where resistance to bacitracin was prevailing, and sediments with dominating multidrug resistance genes (Fang *et al*. 2019). In wastewater samples from swine feed yard, metagenomics approach revealed the presence of ARGs in bacteriophage DNA fraction and the inefficacy of wastewater treatment to induce significant changes in the relative abundance of all bacteria- and phage-associated ARG pool (Wang *et al*. 2018). Moreover, a possible spread of AMR through phage transduction was pointed out by resistance to β-lactam antibiotics and by detection of different plasmid types encoding mobilized colistin resistance (*mcr-1*) gene already described in surface water environments worldwide (Drali *et al*. 2018; Kneis, Berendonk and Heß 2019; Tuo *et al*. 2018; Wang *et al*. 2018; Wang *et al*. 2018). The phage transduction was independent from the type of environment and, in the case of β-lactam resistance, showed a high spreading ability despite the low ARG abundance. Importantly, colistin is considered the last resort defense against multi-drug resistant Gram-negative bacteria (Nation *et al*. 2015). Shotgun sequencing and gene profiling metagenomics confirmed a phage-bacterial interplay in aquaculture wastewater by detecting mobilized ARGs, especially those involved in the modulation of antibiotic efflux pumps, even in the absence of antibiotic treatment (Colombo *et al*. 2016). A small-scale modelling has been adopted to describe the dynamics of phage-mediated AMR spread in less hostile environments where bacterial mortality is low, such as those with sub-inhibitory concentrations of antibiotics and with the presence of ARB (Sankalp *et al*. 2020).

Metagenomic studies on surface water biofilm niches, where HGT is largely facilitated (Madsen *et al*. 2012), are extremely limited and mostly refer to highly competitive environments like wastewaters. In recent years, bacterial membrane vesicles (MV) have been considered as DNA reservoirs at the basis of a new interspecies HGT mechanism in biofilms (Abe, Nomura and Suzuki 2020). Since minimal inhibitory concentrations (MIC) of antibiotics tolerated by biofilm-growing bacteria are conspicuously elevated compared with those applied to planktonic microorganisms (De Oliveira *et al*. 2016; Macia, Rojo-Molinero and Oliver 2014), chronic exposure may enhance mechanisms selecting for resistant phenotypes in these high-density microbial communities (Stewart 2002). Investigations based on shotgun metagenomics suggested that antibiotic pressure of 1 μg L^–1^ may be selective for resistant genotypes (Kraupner *et al*. 2018; Lundström *et al*. 2016), although this concentration falls in the upper range of antibiotic levels reported in European waterbodies (Sanseverino *et al*. 2019). A biofilm matrix allows the concentration of bacterial molecules, such as extracellular DNA released from damaged cells or signaling compounds, and reduces the penetration of exogenous substances (e.g. antibiotics) favoring tolerable exposure to bioactive mixtures. These conditions increase mutation rates and promote plasmid conjugation as well as transformation events through cell-to-cell interactions (Macia, Rojo-Molinero and Oliver 2014; Stadler and Top 2016). Such mobilization of ARGs persisting in a WWTP effluent biofilm was suggested to pose a risk of dissemination to receiving waterbodies as the relative abundance of ARGs associated with MGEs, which in system's effluent reached up to 82% (Petrovich *et al*. 2018) and differed based on the MGE type respect to river biofilms (Balcázar, Subirats and Borrego 2015). Numerous *in vitro* studies demonstrated that sub-inhibitory doses of some antibiotics are sufficient to specifically induce the switch from planktonic to biofilm forming phenotype of bacteria (Hoffman *et al*. 2005; Kaplan 2011). In the Yangtze Estuary, biofilms rather than sediments and water had a major contribution to ARG abundance that was greatly conferred by extracellular DNA (Guo *et al*. 2018).

Metagenomics is a promising technique for ARG mapping in different water environments in terms of relative abundance (detection normalized to total number of reads or more rarely total cell count in a sample), classification (type of conferred resistance) and impact (the extent of ARG propagation) (Chen *et al*. 2019; Li *et al*. 2015) with potential application in risk prediction and assessment of the environmental resistome based on identified ARG profiles (Amos *et al*. 2015). Several AMR clusters have been generated in line with specific anthropogenic pressures on natural waterbodies including low-impact areas where lower ARG richness correlated with minor genetic and microbial diversity (Hong *et al*. 2018; Li *et al*. 2015; Pal *et al*. 2016; Uyaguari-Díaz *et al*. 2018; Yang *et al*. 2019b). However, some technical aspects of shotgun metagenomic sequencing are still to be improved or clarified, including a definition of detection limits for various target genes, generation of short in length and random DNA reads, inadequate quality of databases for sequence identification, selection of the most suitable normalization method in the analysis of data (Pereira *et al*. 2018) along with missing criteria for comparison of data between different platforms (Durso, Miller and Wienhold 2012).

Metagenomics employed to investigate ARG propagation in aquatic ecosystems greatly improves the predictive ability of AMR impact on environmental and human microbiome, the latter defined as the collective genomes of microbes living on and within the human body (Baron, Diene and Rolain 2018; Dang *et al*. 2017; Guo *et al*. 2016). This technique is expected to reduce knowledge gaps in this context, even though the identification of DNA stretches putatively associated with ARGs should rely on verifying whether detected sequences are effectively responsible for conferring antibiotic resistance (Hadjadj *et al*. 2019). Approaches for predictive modeling of resistome analysis have been elaborated to face the question regarding data complexity and low sensitivity/specificity of currently available metagenomics methods (Arango-Argoty *et al*. 2018; Berglund *et al*. 2019; Lanza *et al*. 2018). Similarly, an updated profile of antibiotic resistome has been developed as online searching platform based on available databases confirming that water environments and animal feces cover 75% of all ARG families (Zhang *et al*. 2020).

A recent global prediction of AMR in untreated urban sewage by metagenomics approach revealed geographically specific distribution of abundance and diversity of ARGs that highly correlated with sanitation and health of clustered populations suggesting the improvement of both variables to mitigate the AMR burden worldwide (Hendriksen *et al*. 2019). In the last years, functional metagenomics approaches have been developed for activity-based screening of known and novel ARGs through the generation of metagenomics libraries followed by DNA expression in heterologous hosts, with a potential to identify sequences subjected to HGT (Mullany 2014). Major methodological challenges are linked to technical steps in library construction, sample purity and to the sequence diversity originally present in extracted DNA captured in constructed libraries (Lam *et al*. 2015). Despite always more numerous pieces of evidence supported by outcomes of functional metagenomics applied to river and ocean environments (Amos *et al*. 2014; Hatosy and Martiny 2015; Marathe *et al*. 2018) and a groundbreaking potential of functional gene annotation complementary to sequence-based metagenomics, factors underlying ARG distribution in surface water and sediments remain unclear (Chen *et al*. 2019; Jiang *et al*. 2018; Marathe *et al*. 2018).

Recently, a potential of high-throughput quantitative PCR (HT-qPCR) has been highlighted in the evaluation of ARGs in various environmental matrices, as a cost-effective technology requiring low sample volumes and less complex bioinformatic tools than metagenomics, which results particularly relevant for routine AMR analysis. Nonetheless, HT-qPCR is based on primers specific to the assessed sequences for which individual protocols must be optimized, therefore metagenomics remains the solution of choice for the identification of hitherto unknown resistance genes in research-oriented analysis (Waseem *et al*. 2019).

Technological advancements along with improved data analytics platforms seen in recent years provide always more sophisticated means for AMR detection and prediction (Gupta, Tiwari and Cytryn 2020). Not free from drawbacks, each method adapts to specific purposes such as routine analysis requiring low costs and straightforward protocols or environmental modeling in support of decision making based on high throughput techniques, allowing processing a large number of samples simultaneously and detecting a large number of ARGs per sample.

## FINAL REMARKS

The universal interconnection between humans, biota and environment constitutes the axis of the One Health concept seeing well-being of each compartment dependent on mutual equilibrium. A better understanding of AMR selection pressures and ARG propagation is needed for an efficient and reliable risk assessment that ARB may pose on human and animal health. This can be achieved through increased scientific efforts to identify metabolic pathways inducing antibiotic resistance in bacterial species (e.g. basic cell metabolism modified by mutations in ATP synthase subunits) (Zalis *et al*. 2019).

Although the importance of standardized surveillance methods has been pointed out by several authors (Ben *et al*. 2019; Matheu, Aidara-Kane and Andremont 2017; Berendonk *et al*. 2015; Huijbers, Flach and Larsson 2019; Larsson *et al*. 2018), guidance taking into account both the toxicity and the acquisition of resistance by microorganisms in the environmental context is missing.

**Figure 2.**
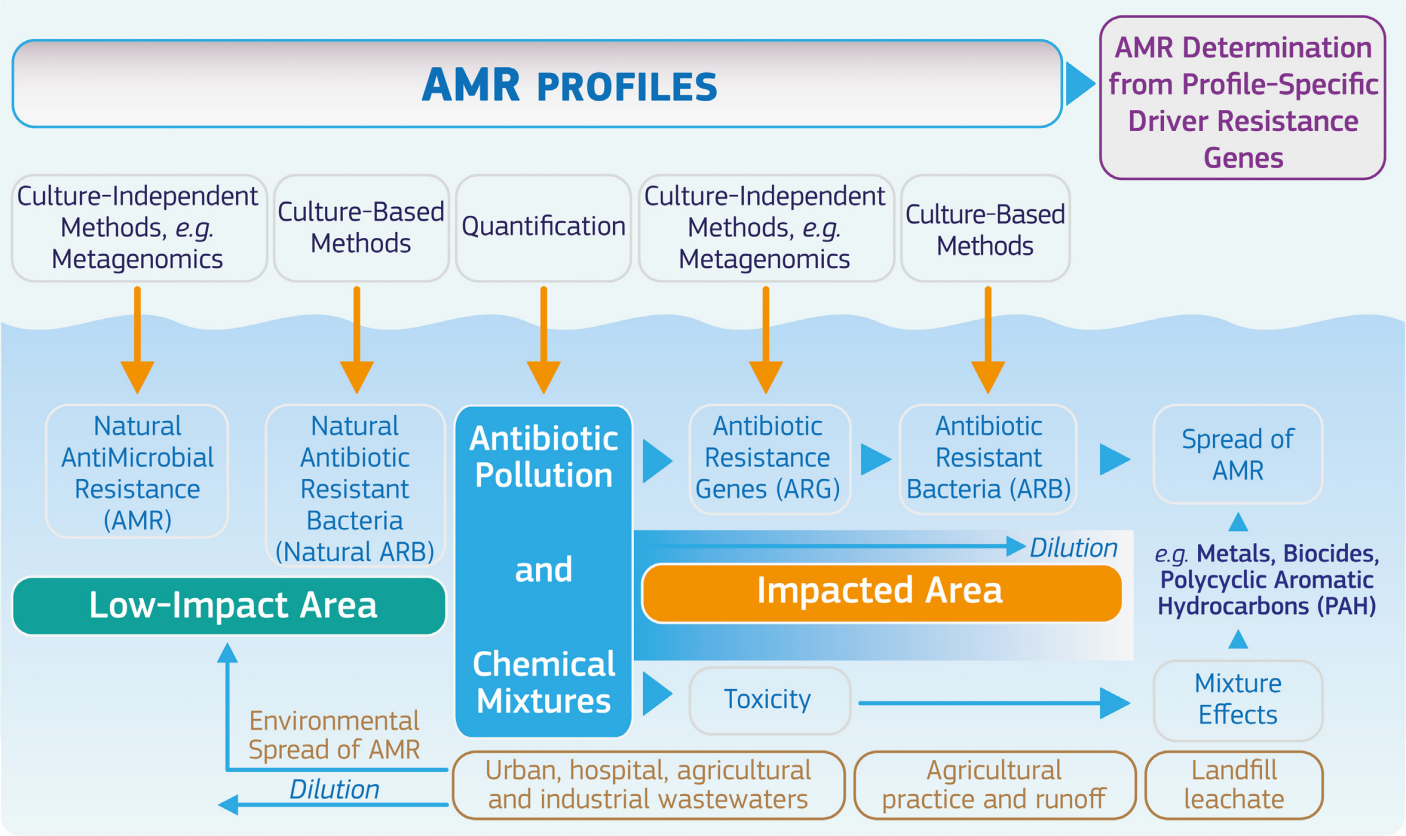
AMR risk assessment and mapping platform. The evaluation of effects induced by antibiotics in aquatic organisms may be evaluated in concert with detection and activity of ARGs through application of culture-based and culture-independent methods in low-impact and high-impact areas. ARG mapping for each type of pollution performed by means of metagenomics may help in generating AR profiles with specific driver antibiotics. Dilution factor due to site's natural characteristics and the contribution of chemical pollutants (e.g. metals, biocides, polycyclic aromatic hydrocarbons (PAH)) must be considered with particular attention for the AMR spread as the stability of ARGs may not be reflected by correlation with concentrations of antimicrobials.

Functional metagenomics is a paramount approach to fill the knowledge gaps (Table 1) regarding correlation between the presence and the functional role of ARGs along with the definition of minimal concentrations of antibiotics able to induce AMR. Generation of ARG profiles in water environments and specific to antibiotic pressures in differentially impacted areas would have a great advantage in strategic planning for the mitigation of AMR effects (Fig. 2). However, the contribution of bioactive antibiotic metabolites and other compounds known for their co-selection potential must be considered based on contamination profiles of chemical mixtures in waterbodies. Also, the anthropogenic footprint in formerly pristine areas due to environmental transport of AMR determinants requires reconsideration of reference habitats used as controls for scientific studies and definition of low-impact areas.

**Table 1.** Major knowledge gaps and technical limitations in the environmental assessment of AMR.

Issue	Current approach	Knowledge gap	Solution
AR detection	Culture-based methods Culture-independent methods (excluding metagenomics) Metagenomics	Not representative for natural microbial communities and environmental conditions, no information on ARG in non-culturable bacteria No information on ARG functional activity Complex data analysis, low comparability between different platforms	Approach combining all methods should be adopted and combined with metagenomics for ARG identification and function Standardization of methods and protocols in ARG metagenomics studies
Environmental concentrations of antibiotics	Studies on samples from sites differently impacted by release of antibiotics or experimental set-ups	Insufficient number of studies reporting correlations between antibiotic concentrations and AMR in surface water	More studies on possible correlations between AMR and antibiotic concentrations unrelated to WWTP
Risk assessment	Safety thresholds based on antibiotic toxicity Bioactive metabolites of antibiotics ARG/ARB distribution and transmission	No standardized approach, no safety threshold for antibiotics considering AMR Not included in risk assessment of antibiotics Incomplete knowledge relative to environmental pathways and affecting factors	Standardization of risk assessment procedure Quantification of bioactive metabolites in risk assessment, more studies on possible effects More studies required
Natural background resistance	Studies on samples from low-impact areas	Few studies, unknown contribution to AMR spread	More studies needed to analyze the selective pressure promoted by antibiotics in low-impact areas
Metal/antibiotic co-resistance	Identification of ARG/MRG and determination of antibiotic/metal concentrations Contribution of microplastics	Unknown mechanisms and concentrations of antibiotics/metals inducing co-resistance Few studies, especially in freshwater	More studies to address the molecular mechanisms involved in the association between metal exposure and the spread of ARG More studies required

**AMR**: antimicrobial resistance; **ARB**: antibiotic resistant bacteria; **ARG**: antibiotic resistance genes; **MRG**: metal resistance genes; **WWTP**: wastewater treatment plant

Methodological approach for environmental assessment and monitoring should avoid evaluation of antibiotics as individual substances but consider molecular interactions within the mixture. More studies are therefore needed to understand the impact of antimicrobials in realistic scenarios including environmentally relevant concentrations of antibiotics and possible additive/synergistic effects of co-occurring compounds on AMR spread also in long-term perspective. For this purpose, technical harmonization and optimization of detection methods are of paramount importance for future regulatory applications aimed at protecting the environment from adverse effects from pharmaceuticals and at the same time ensuring safe and effective use of antimicrobials for human needs. These aspects have recently been discussed during the first Workshop on Pharmaceuticals in the Environment within the EU strategic approach (EC COM(2019a) 128) which gathered unprecedentedly high number of European participants making this event a milestone for further approach on AMR issue at international level. Actions to better understand the link between the presence of antibiotics in the environment and AMR spread have been recommended under the European Union Strategic Approach to Pharmaceuticals in the Environment (EC 2020d). Furthermore, since AMR is an increasing worldwide problem which requires broad scale responses as evidenced by the WHO Global Action Plan (WHO 2015) and the United Nations Interagency Coordination Group (IACG 2019 and 2020), a global and prompt collaboration between all countries in AMR prevention and control is envisaged.
